# Transcriptome-Wide Insights: Neonatal Lactose Intolerance Promotes Telomere Damage, Senescence, and Cardiomyopathy in Adult Rat Heart

**DOI:** 10.3390/ijms26041584

**Published:** 2025-02-13

**Authors:** Olga V. Anatskaya, Sergei V. Ponomartsev, Artem U. Elmuratov, Alexander E. Vinogradov

**Affiliations:** 1 Institute of Cytology RAS, Saint-Petersburg 194064, Russia; ponomartsevsv@gmail.com (S.V.P.); aevin@incras.ru (A.E.V.); 2 Institute of Biomedical Chemistry RAS, Moscow 119121, Russia; artem@genotek.ru

**Keywords:** neonatal lactose intolerance, developmental programming, telomere fusion, DNA damage, senescence, cardiovascular disease, polyploidy, cardiomyocyte, metabolic deprivation

## Abstract

Cardiovascular diseases (CVD) are the primary cause of mortality globally. A significant aspect of CVD involves their association with aging and susceptibility to neonatal programming. These factors suggest that adverse conditions during neonatal development can disrupt cardiomyocyte differentiation, thereby leading to heart dysfunction. This study focuses on the long-term effects of inflammatory and oxidative stress due to neonatal lactose intolerance (NLI) on cardiomyocyte transcriptome and phenotype. Our recent bioinformatic study focused on toggle genes indicated that NLI correlates with the switch off of some genes in thyroid hormone, calcium, and antioxidant signaling pathways, alongside the switch-on/off genes involved in DNA damage response and inflammation. In the presented study, we evaluated cardiomyocyte ploidy in different regions of the left ventricle (LV), complemented by a transcriptomic analysis of genes with quantitative (gradual) difference in expression. Cytophotometric and morphologic analyses of LV cardiomyocytes identified hyperpolyploidy and bridges between nuclei suggesting telomere fusion. Transcriptomic profiling highlighted telomere damage, aging, and chromatin decompaction, along with the suppression of pathways governing muscle contraction and energy metabolism. Echocardiography revealed statistically significant LV dilation and a decrease in ejection fraction. The estimation of survival rates indicated that NLI shortened the median lifespan by approximately 18% (*p* < 0.0001) compared with the control. Altogether, these findings suggest that NLI may increase susceptibility to cardiovascular diseases by accelerating aging due to oxidative stress and increased telomere DNA damage, leading to hyperpolyploidization and reduced cardiac contractile function. Collectively, our data emphasize the importance of the early identification and management of neonatal inflammatory and metabolic stressors, such as NLI, to mitigate long-term cardiovascular risks.

## 1. Introduction

Cardiovascular diseases (CVD) are the leading cause of morbidity and mortality in aging populations, with prevalence rising from ~40% in individuals aged 40–59 to nearly 85% in those over 80 [1,2,3]. Increasing evidence implicates premature cardiac aging as a key risk factor for CVD, underscoring the need to identify its molecular triggers [4,5].

Neonatal programming is a critical determinant of lifelong cardiac health, as early postnatal development coincides with heightened vulnerability to adverse conditions such as malnutrition, oxidative stress, and inflammation [6,7,8,9,10]. Epidemiological studies from Germany, China, and Russia reveal that early-life adversities, including famine and infections, increase the risk of CVD in adulthood, including hypertension, coronary artery disease, and heart failure [11,12,13]. Moreover, extensive epidemiological data from cohorts born before 1900 highlight the role of infectious and inflammatory diseases encountered in infancy and childhood in the development of CVD in old age [14,15]. Experimental models further demonstrate that early-life stressors induce irreversible cardiomyocyte alterations, predisposing individuals to premature cardiovascular dysfunction [16,17,18].

This vulnerability arises because early postnatal development is characterized by significant anatomical and biochemical changes in the heart [19]. These include the closure of fetal shunts (e.g., foramen ovale), metabolic reprogramming to aerobic metabolism, myofibril accumulation, and a transition from proliferative to hypertrophic growth, often accompanied by cardiomyocyte polyploidization [9,10]. In cardiomyocytes, polyploidy is accompanied by the loss of the ability for mitotic division and a shift from proliferative growth to hypertrophy [20,21,22,23]. Stabilizing at a certain level during postnatal growth, the number of genomes in human and other mammalian cardiomyocytes largely reflects the events of postnatal ontogenesis, because polyploidy increases easily in response to various stressful conditions and insults [23,24,25,26].

It is well established that polyploidy plays an important role in regulating adaptive and pathological processes and is associated with a wide range of cardiovascular diseases, including cardiomyopathies of various origins, congenital heart defects, hypertension, ischemic heart disease, and myocardial infarction [27,28]. High variability in cardiomyocyte ploidy among individuals is notable, with some predominantly exhibiting cells with 2–4 genomes, while others have 8–16 [24,27,29]. Considering that polyploidization is a marker of cardiovascular diseases and is associated with a decline in the functional and regenerative potential of the heart [28,30], it is reasonable to suggest that healthy individuals with increased cardiomyocyte ploidy are at higher risk for developing subclinical and clinical cardiovascular pathologies compared with those with normal cardiomyocyte ploidy.

Based on these observations, we propose that excessive polyploidy can play a role in CVD development associated with neonatal programming and premature aging. Identifying triggers of excessive cardiomyocyte polyploidization and understanding the resulting disruptions in regulatory and metabolic networks are crucial for advancing preventive and therapeutic strategies for cardiovascular diseases.

This study is a continuation of our previous work investigating the long-term effects of NLI on systemic inflammatory stress, cardiomyocyte remodeling, and transcriptomic changes in rats [31]. Earlier, we focused on “toggle genes”—genes expressed exclusively in either the control or experimental groups—as markers of profound cellular changes [31]. These genes provided insights into the physiological and molecular factors driving long-term alterations [32,33].

In this study, we focus on genes with quantitative expression changes in response to NLI, particularly those regulating energy metabolism, cell cycle signaling, stress responses, and transport. We also expand our analysis of cardiomyocyte polyploidy across multiple left ventricular (LV) regions and assess cardiac function and survival using echocardiography and Kaplan–Meier analysis.

Our findings reveal that NLI induces excessive cardiomyocyte polyploidization and nuclear bridging across LV regions. Transcriptomic analysis identified the activation of DNA damage response in telomeric regions, chromatin remodeling, and pathways associated with aging, cardiomyopathy, and hypertension. Correspondingly, NLI reduces the expression of genes essential for energy metabolism and muscle contraction, contributing to LV dilation and impaired cardiac function. Survival analysis further demonstrated an NLI-associated reduction in median lifespan by ~18%, highlighting its profound impact on cardiac aging and disease susceptibility.

## 2. Results

### 2.1. Assessment of Survival Rate

Assessing survival rates is a fundamental approach in biomedical research for evaluating the long-term impact of early-life interventions on lifespan and aging processes. By analyzing survival curves, it is possible identify factors that accelerate age-related decline, providing insights into potential mechanisms underlying premature mortality. In this study, neonatal NLI was examined for its effects on lifespan in rats.

**Figure 1:**
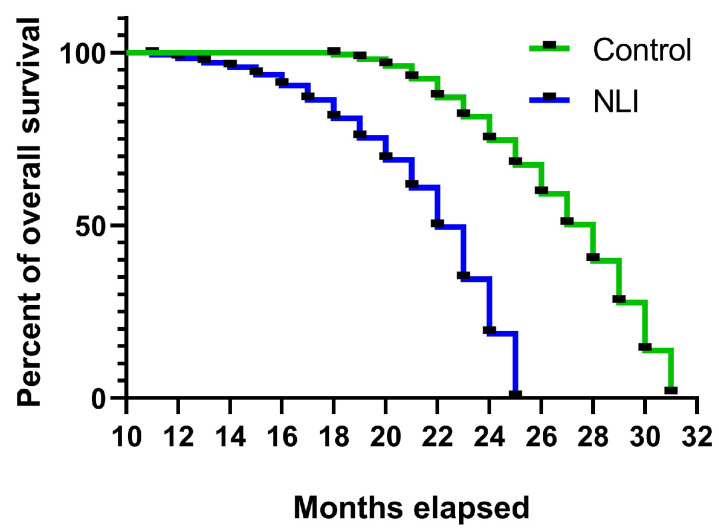
The Kaplan–Meier curves of survival rates for NLI rats and control rats. NLI-survived rats show reduced lifespan compared with control (*p* < 0.0001; Mantel-Cox test).

Figure 1 presents the survival curves for rats neonatally treated with lactose (*n* = 12) compared with control rats (*n* = 12). The data clearly demonstrate that rats treated with lactose (NLI) had a significantly shorter lifespan than the control group (*p* < 0.0001). The median survival age for the lactose-treated group was approximately 23 months, whereas the control rats had a median survival age of 28 months. This represents an 18% reduction in the median lifespan for the lactose-treated rats. The last lactose-treated rat died at 25 months, at which point 30% of the control group was still alive. The last control rat survived until 31 months, further highlighting the longevity disadvantage conferred by neonatal lactose intervention.

### 2.2. Echocardiography

**Figure 2:**
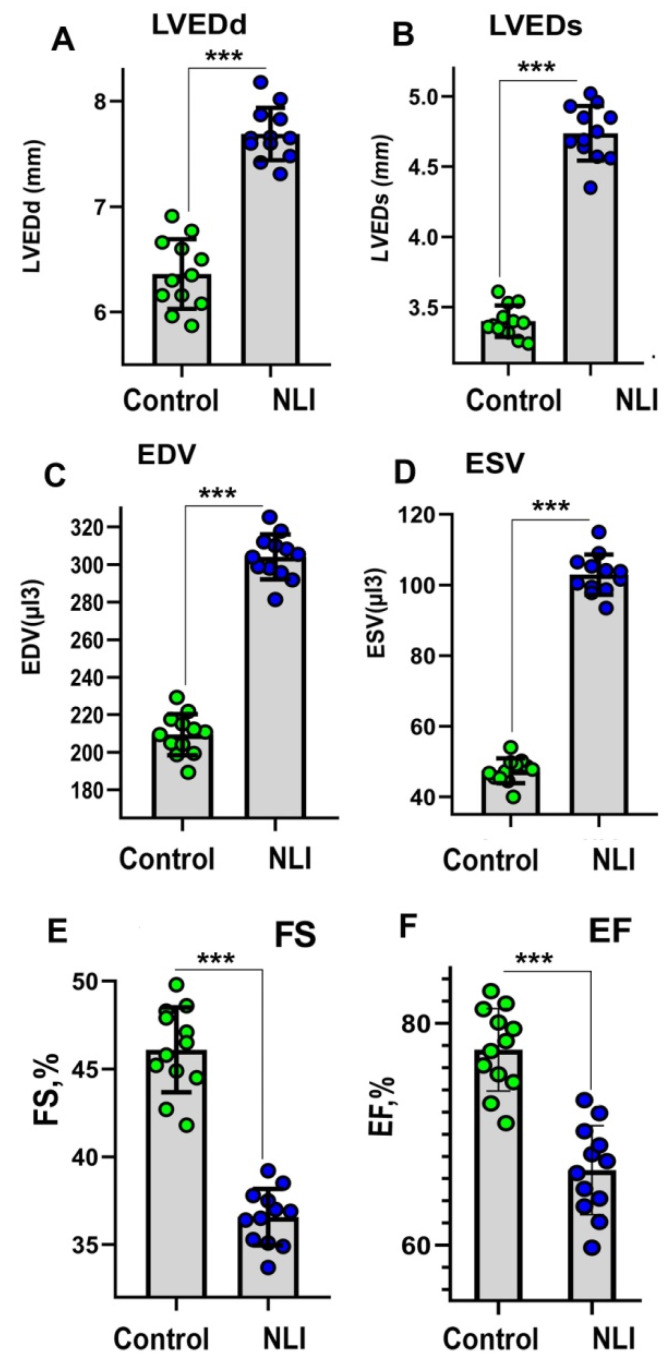
Echocardiography analysis of the hearts from NLI-survived and control rats at the age of 140 days. (**A**)—left ventricle end diastolic diameter (LVEDd); (**B**)—left ventricle end systolic diameter (LVEDs); (**C**)—end diastolic volume (EDV); (**D**)—end systolic volume (ESV); (**E**)—fraction shortening (FS); (**F**)—ejection fraction (EF). Green symbols show control, Blue symbols show the Experiment. ***—*p* < 0.001.

To evaluate the long-term effects of NLI on cardiac performance, we assessed left ventricular (LV) geometry and function using conventional echocardiography. At 140 days of age, the impact of NLI remained evident (Supplementary Table S1). Figure 2 highlights parameters demonstrating statistically significant differences between the experimental and control groups. The most prominent anatomical changes included marked increases in left ventricular end-diastolic and end-systolic diameters (LVEDd and LVESd, respectively), accompanied by corresponding enlargements in end-diastolic and end-systolic LV volumes (EDV and ESV). These structural alterations led to impaired LV function, evidenced by a reduction in ejection fraction (EF) exceeding 15% and a nearly 20% decrease in fractional shortening (FS) (Supplementary Table S1, Figure 2).

Altogether, these NLI-induced changes are characteristic of the maladaptive remodeling of the myocardium, suggesting the existence of cardiomyopathy with signs of LV dilatation. The signs of LV dilation were seen from the increase in end-systolic and end-diastolic LV diameters and volumes and from the decrease in EF and FS [34]. At the same time, although we identified statistically significant differences between the experimental and control groups, it is important to note that we are dealing with a subclinical, mild form of cardiomyopathy. In clinical forms, the differences between health and diseases are much more pronounced and typically exceed 35–50% in these parameters [35,36,37]. In this experiment, the maximum differences rarely exceeded 20% (Supplementary Table S1).

### 2.3. Analysis of Cardiomyocyte Ploidy in Different Regions of the Left Ventricle

**Figure 3:**
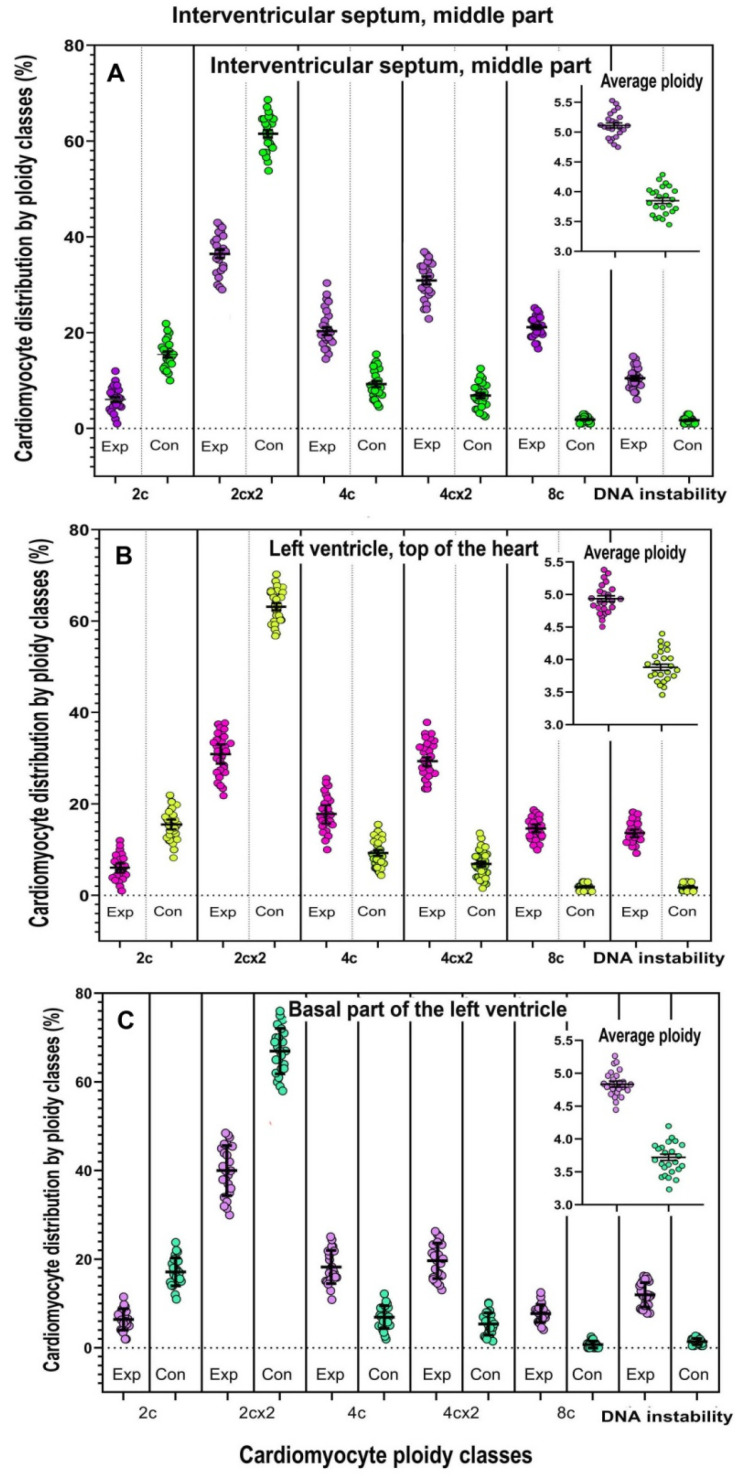
Distribution of cardiomyocytes by ploidy classes in different regions of the cardiac left ventricle, including the interventricular septum (**A**), apex (**B**), and basal part (**C**). It is evident that polyploid cells are present in all regions of the left ventricle, with increased ploidy primarily resulting from the accumulation of tetraploid and octoploid nuclei. Control and the Experiment are shown in different colors.

To validate the association between NLI and increased cardiomyocyte ploidy across the left ventricle (LV), we assessed polyploidy prevalence at three regions: the tip of the cardiac apex, the basal part of LV, and the midsection of the interventricular septum. Cytophotometry confirmed elevated cardiomyocyte ploidy in experimental animals compared with controls. This increase predominantly resulted from a higher number of mononuclear tetraploid and octoploid cells, with the latter exhibiting either a single octoploid nucleus or two tetraploid nuclei (Figure 3). Only relatively minor gradient differences were observed: basal part (4.7 ± 0.4) → apex (4.9 ± 0.4) → septum (5.2 ± 0.5) in the experimental group, and a similar gradient in the control group: basal part (3.7 ± 0.2) → apex (3.8 ± 0.2) → septum (4.0 ± 0.4) (Figure 3).

### 2.4. Transcriptome Changes in the Left Ventricle (LV) of the Heart in Adult Rats After NLI

**Figure 4:**
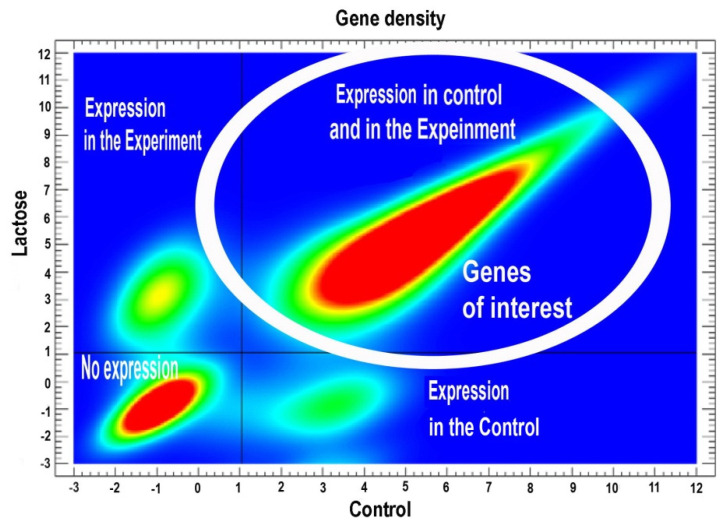
Two-dimensional histogram of gene expression levels in the experiment (Lactose) and control (Control). Four density peaks in the gene expression distribution are visible: the **bottom**–**left** (no expression in either the experiment or control), **top**–**left** (expression only in the experiment), **bottom**–**right** (expression only in the control), and **top**–**right** (expression in both the experiment and control—the peak of primary interest). Blue to red color gradient reflects positive gradient in gene density (the lowest density is shown in blue, the highest density is shown in red). A white circle shows genes of interest, i.e., the genes demonstrating quantitative difference between control and the experiment.

In this study, we focused on differentially expressed genes (DEGs) with gradual changes in expression. These genes were chosen because they are typically involved in essential cellular processes and cannot be completely turned off or on. Furthermore, the analysis of these genes complements and extends the results obtained previously for switched-on/off genes [31]. To identify these genes, we selected the ones that exhibited quantitative changes in expression, meaning they were expressed both in the control and under experimental conditions but at different levels of intensity. For this purpose, we considered the density peaks of genes on a two-dimensional histogram as a criterion for the distribution of gene expression in the control and experimental conditions. The gaps between the peaks indicate qualitative (discrete) changes in expression, corresponding to transitions between different states, i.e., the switching on/off of genes. The genes of interest fall within the largest cloud located at the upper right part of the two-dimensional histogram presented at Figure 4.

**Figure 5:**
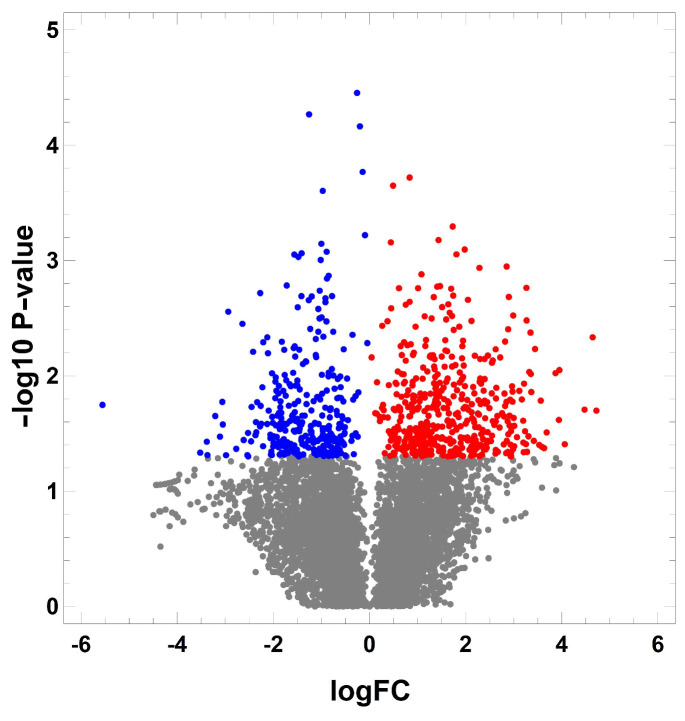
Transcriptome changes in the left ventricle (LV) of the heart in adult rats after NLI (volcano plot). Only genes with quantitative changes in expression are shown, meaning those expressed in both the control and experimental groups. log FC represents the logarithmic values of expression differences between the control and experimental groups. log10 *p*-value indicates the statistical significance of these differences. Gray dots represent non-significant changes, red dots indicate increased expression (*p* < 0.05), and blue dots indicate decreased expression (*p* < 0.05).

Figure 5 presents a volcano plot showing the relationship between gene expression differences and their statistical significance for genes that only exhibited changes in expression, i.e., DEGs expressed in both control and experimental conditions.

To identify functional clusters among these genes, we applied the “Glay Clustering” algorithm implemented in Cytoscape. For the analysis, we selected only large clusters containing more than 20 genes. Among the NLI-induced DEGs, Glay clustering identified four major clusters related to telomere maintenance (59 genes), senescense (40 genes), and macroautophagy (35 genes), and the complement system (120 genes). Among DEGs with reduced expression, three major clusters were identified, associated with cell cycle and differentiation (139 genes), muscle contraction (30 genes), energy metabolism (63 genes), and immunity (59). The NLI up- and downregulated genes comprising Glay clusters are presented in Supplementary Tables S2 and S3.

Each cluster was further analyzed using the Metascape server [38], which identifies molecular complexes and their biological features. Below, we provide a detailed description of these gene clusters.

### 2.5. NLI Is Associated with the Disruption of Telomere Maintenance, Chromatin Opening, and Genetic Instability

**Figure 6:**
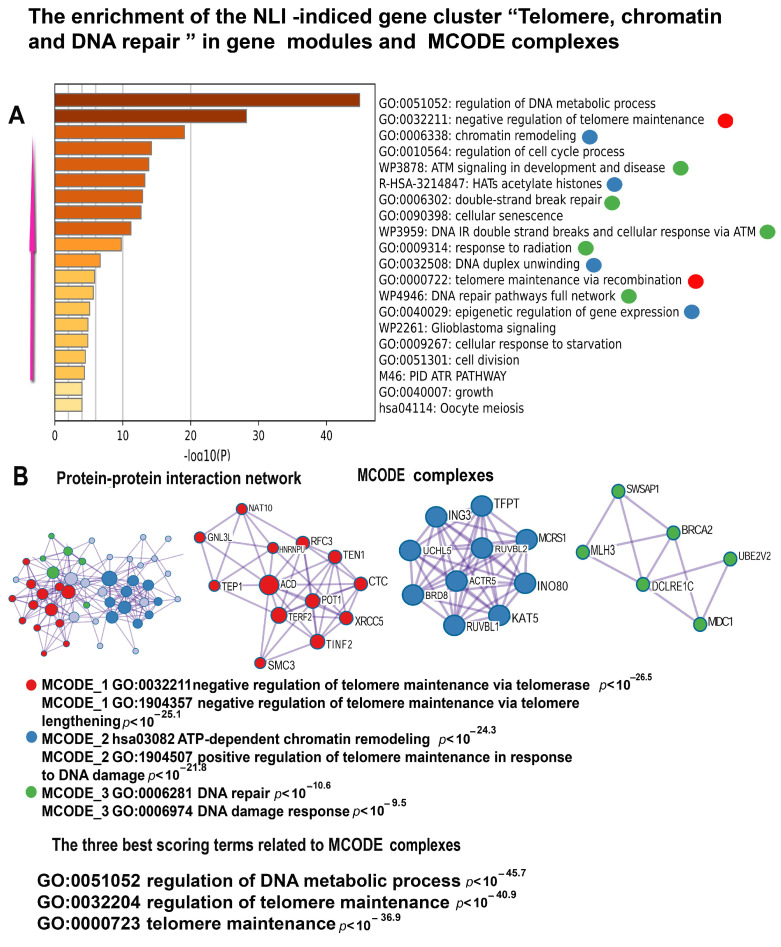
Enrichment of NLI-induced genes related to the “Telomeres, Chromatin, and DNA Repair” cluster in gene pathways, processes, and molecular complexes. (**A**)—Histogram of gene modules and biological processes. The statistical significance of enrichment is shown on the X-axis (−log10(*p*)). (**B**)—Protein–protein interaction network and MCODE components (i.e., densely connected network components). The network and MCODE components were constructed based on physical interactions obtained from the STRING server (physical score > 0.4). The three most significant terms related to the MCODE components are highlighted. The coding by color squares reflects the MCODE components. The coding by color circles indicates the results of the MCODE component pathway and process enrichment analysis. The figure illustrates the association between NLI and long-term telomere DNA and telomere damage, as well as chromatin remodeling.

Among the NLI upregulated genes, the most important cluster is related to telomere biology, DNA damage response, and chromatin remodeling (Figure 6). Below, we provide a detailed functional description of gene modules and molecular complexes related to these functional categories.

#### 2.5.1. Telomere Maintenance

Two gene modules emerge as particularly significant for telomere regulation: “negative regulation of telomere maintenance via telomerase” (*p* < 10⁻^22^) and “telomere maintenance via recombination” (*p* < 10⁻^4^). The first module highlights diminished telomerase activity, leading to progressive telomere shortening and dysfunction. This, in turn, results in telomere instability, a phenomenon well-recognized in the literature as a driving factor in various age-related diseases, including cardiovascular diseases, cancer, and other degenerative conditions [39]. These findings underscore the critical role of telomere regulation in maintaining genomic integrity and its implications for health and disease.

The second module involves the maintenance of telomere length through recombination, a critical mechanism of the alternative lengthening of telomeres (ALT). This pathway is particularly significant in telomerase-negative tumors, where it has been strongly implicated in promoting carcinogenesis [40]. Genes within this module often show increased activity in response to DNA damage at telomere regions, emphasizing the interconnected roles of DNA repair pathways and telomere dynamics [40,41]. These observations point to a direct role of NLI in compromising telomere integrity. By disrupting pathways essential for telomere maintenance, such as ALT and telomerase regulation, NLI could lead to genomic instability. This instability not only predisposes cells to age-related diseases like cardiovascular dysfunction but also facilitates oncogenesis in cells maintaining telomere recombination mechanisms [42]. These findings underscore the urgent need for further research into the biological and clinical consequences of NLI, particularly its effects on genomic stability and disease susceptibility.

#### 2.5.2. Epigenetic Regulation and Chromatin Remodeling

The gene modules related to epigenetic regulation include “histone acetyltransferases (HATs) acetylating histones”, “DNA duplex unwinding”, “chromatin remodeling”, “protein localization to the chromosome” (*p* < 10^−12^ for all), and “epigenetic regulation of gene expression” (Figure 6). Notably, the first two modules highlight processes fundamental to chromatin opening—histone acetylation and DNA unwinding—indicating that these molecular events may drive extensive chromatin remodeling. Such large-scale chromatin reorganization likely amplifies transcriptional dynamics and disrupts cellular homeostasis, further contributing to disease phenotypes.

#### 2.5.3. DNA Damage Response and ATM Signaling

The association between NLI and DNA instability seen from gene modules involved in the DNA damage response are presented by pathways like “ATM signaling in development and diseases” (*p* < 10⁻^15^), “double-strand break repair” (*p* < 10⁻^12^), and “DNA damage-induced double-strand breaks and cellular response via ATM” (*p* < 10⁻^12^). The central role of ATM signaling in these pathways underscores its importance in orchestrating cellular responses to double-strand breaks (DSBs), the most cytotoxic form of DNA damage [43]. ATM kinase activates a network of repair proteins critical for maintaining genomic stability.

The “double-strand break repair” module emphasizes homologous recombination and non-homologous end joining—two essential mechanisms for resolving DSBs. Persistent activation of ATM-dependent pathways by NLI could lead to chronic DNA damage signaling, reshaping the gene expression landscape and further impairing cellular function. This prolonged activation may also synergize with disruptions in telomere maintenance and chromatin remodeling, compounding the risk of genomic instability and associated diseases [44]. Supporting the main findings of gene functional enrichment analysis, the MCODE analysis revealed three molecular complexes related to the biology of telomeres. The first and the most important complex is “negative regulation of telomere maintenance via telomerase” (GO: 0032211, red symbols), unifying 13 genes (Supplementary Table S2, Figure 6). Among these genes, there are several crucial regulators of telomere maintenance, repair, recombination, and length regulation. Thus, GNL3L and NAT10 induce telomere shortening [45] whereas HNRNPU and TINF2 were reported to negatively regulate telomere length [46]. The genes MDC1, CTC1, XRCC5, RFC3, SMC3, UBE2V2, TEP1, CDK2, TERF2, and POT1 participate in telomere protection and subthelomeric DNA repair. In addition, several of these genes (SMC3, TEP1, POT1, and TERF2) can also participate in alternative telomere lengthening (ALT) [47,48]. The second complex is enriched in genes from a GO biological processes “positive regulation of telomere maintenance in response to DNA damage” (GO:1904507) and “positive regulation of DNA repair” participating in DNA repair in the subtelomeric region. This complex is also enriched in genes form a GO pathway “ATP-dependent chromatin remodeling” that promotes chromatin opening, thereby facilitating the access of DNA repair enzymes to sites of DNA damage [49]. The third molecular complex unifies genes from the pathway “Polymerase switching on the C-strand of the telomere” involved in telomere lengthening [50]. Altogether, the analysis of the telomere-related gene cluster suggests that NLI can stimulate ALT to compensate for the deficiency of TERT-dependent telomere lengthening. This is evident from the gene pathway “negative regulation of telomere maintenance via telomerase” and from the induction of gene modules related to DNA recombination and repair in telomeric and subtelomeric region that are known to promote ALT [51].

#### 2.5.4. The Other Gene Modules in the Telomere Related Cluster

Additional gene modules within this cluster, although of lower statistical significance, continue to reinforce key functional themes related to double-strand break repair, chromatin remodeling, and telomere maintenance (Figure 6). The only exception is the “Cellular Aging” module, which does not completely align with these core functional categories. However, its connection to other modules is likely mediated through shared pathways such as DNA damage, telomere shortening, and chromatin alterations—all of which are major drivers of aging [52]. The inclusion of the “Cellular Aging” module in this cluster suggests its potentially broader biological significance. It highlights the link between genomic instability and the progressive disruption of cellular homeostasis and functionality, hallmark features of aging and age-related diseases. Thus, NLI may exert multifaceted effects on cellular aging by modulating key molecular pathways.

#### 2.5.5. Morphological Signs of Telomere Damage and Fusion

**Figure 7:**
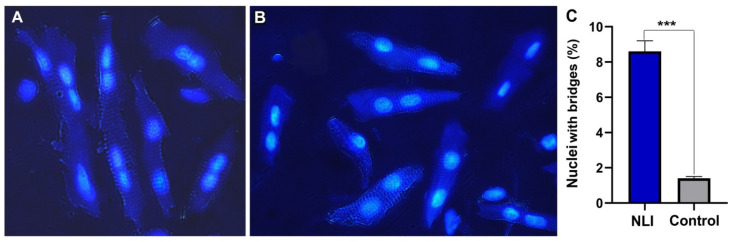
**Isolated cardiomyocytes with joined nuclei as an indicator of telomere fusion** (**A**)—cardiomyocytes with joined nuclei form the heart of an experimental animal. (**B**) cardiomyocytes with separated nuclei from the control animal. Cells were isolated with enzymatic method, nuclear staining with Hoechst 33258. Combined transmitted light and fluorescence imaging. Phase contrast, total magnification X200. (**C**) Bar chart illustrating the difference in the percentage of joined nuclei between the experimental and control animals. ***—*p* < 0.00001, binomial proportion. Blue color– indicates the experiment; Grey color indicates control.

One of the morphological signs of telomere fusion is the appearance of double, dumbbell-shaped nuclei [40]. Such nuclei arise due to abnormalities in nuclear division caused by the formation of telomeric DNA bridges. To morphologically validate bioinformatics data on DNA damage in the telomere regions, we analyzed the prevalence of double nuclei in 1000 cardiomyocytes from three experimental and three control animals, using smears of isolated cardiomyocytes. Our data revealed a significantly higher number of double nuclei in the experimental animals compared with the control group. For isolated cardiomyocytes, the difference reached about 6 fold (8.5% ± 0.6 in the experiment versus 1.4% ± 0.1 in the control group, *p* < 0.00001, and the binomial test for isolated cells (Figure 7A–C)). Interestingly, cells with double nuclei were unevenly distributed in isolated cell smears. In some visual fields, these cells predominated over normal ones (Figure 7A).

#### 2.5.6. NLI-Downregulated Genes Reveal Cell Cycle Disruption via M-Phase Restitution and Confirm Chromatin Decompaction and Impaired Differentiation

**Figure 8:**
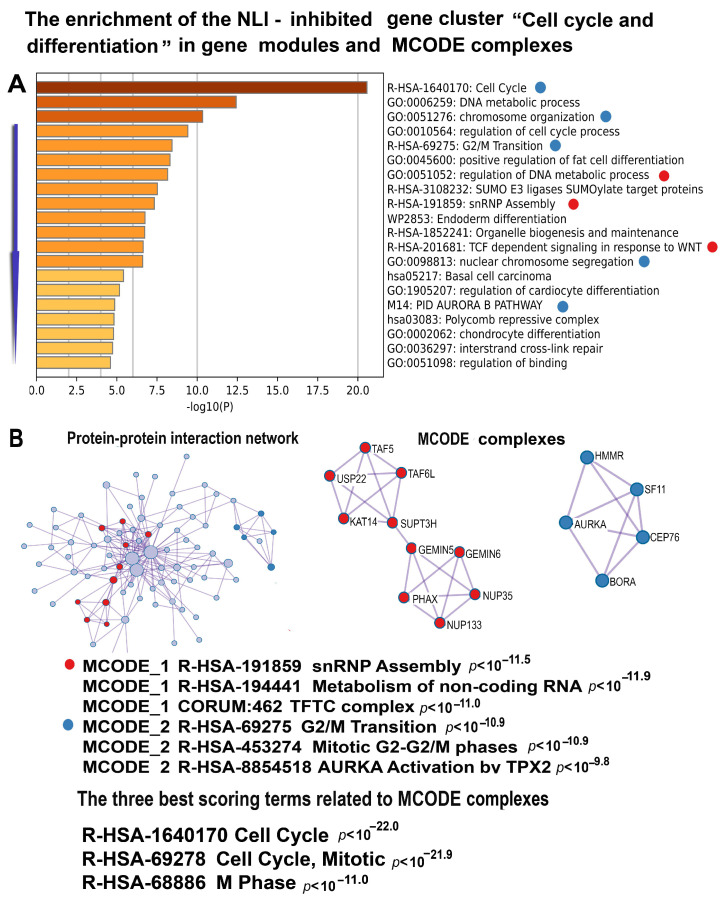
Enrichment of NLI-inhibited genes related to the “Cell Cycle and differentiation” cluster in gene pathways, processes, and molecular complexes. (**A**)—Histogram of gene modules and biological processes. The statistical significance of enrichment is shown on the X-axis (−log10(*p*)). (**B**)—Protein–protein interaction network and MCODE components (i.e., densely connected network components). The network and MCODE components were constructed based on physical interactions obtained from the STRING server (physical score > 0.4). The three most significant terms related to the MCODE components are highlighted. The coding by color squares reflects the MCODE components. The coding by color circles indicates the results of the MCODE component pathway and process enrichment analysis. The figure illustrates the association between NLI and long-term cell cycle restitution and the impairment of cell differentiation.

Reinforcing the data presented by the telomere-related gene cluster of upregulated genes and the morphological confirmations of telomere fusions (Figure 6 and Figure 7), the NLI downregulated genes contain a functional cluster confirming cell cycle disruption by the M-phase restitution, chromatin decompaction, and impaired differentiation (Figure 8). Thus, gene functional enrichment analysis detected gene modules “Cell Cycle,” “Chromosome segregation”, ”Chromosome organization”, and “G2/M transition” as the most significantly enriched pathways. Additionally, MCODE clustering identified a molecular complex implicated in G2/M transition and cell cycle regulation (Figure 8). These results suggest that disruptions in the G2/M transition underline a broader dysregulation of the cell cycle. Such mitotic rearrangements are suggestive of polyploidy, potentially arising from the failure of late mitotic stages, a phenomenon described as restitutional mitosis [53,54,55,56,57,58].

The cluster of downregulated genes further revealed signs of chromatin relaxation, evident from the suppression of key nucleosome components, including histones H4, H2, and H3 families (Supplementary Table S3). This pattern of chromatin loosening aligns with evidence of chromatin opening processes, such as histone acetylation and DNA duplex unwinding, observed within the upregulated telomere-related gene cluster (Figure 6).

It is also important to note that the cell cycle-related gene cluster is enriched in several gene modules related to cell differentiation, including “Regulation of cardiocyte differentiation”, “Chondrocyte differentiation”, and “Regulation of fat cell differentiation”. This result is in good agreement with the data indicating that telomere shortening impairs cardiomyocyte differentiation potential by shifting phenotype towards a more smooth muscle cell-like identity in the cells [59].

Taken together, these results suggest that NLI may have long-term detrimental effects on telomere and chromatin integrity. By disrupting key pathways involved in telomere and chromatin maintenance, NLI could contribute to genomic instability and increase susceptibility to telomere dysfunction-related heart diseases.

### 2.6. NLI Activates Signaling Cascades Related to Senescence and Cardiomyopathy

**Figure 9:**
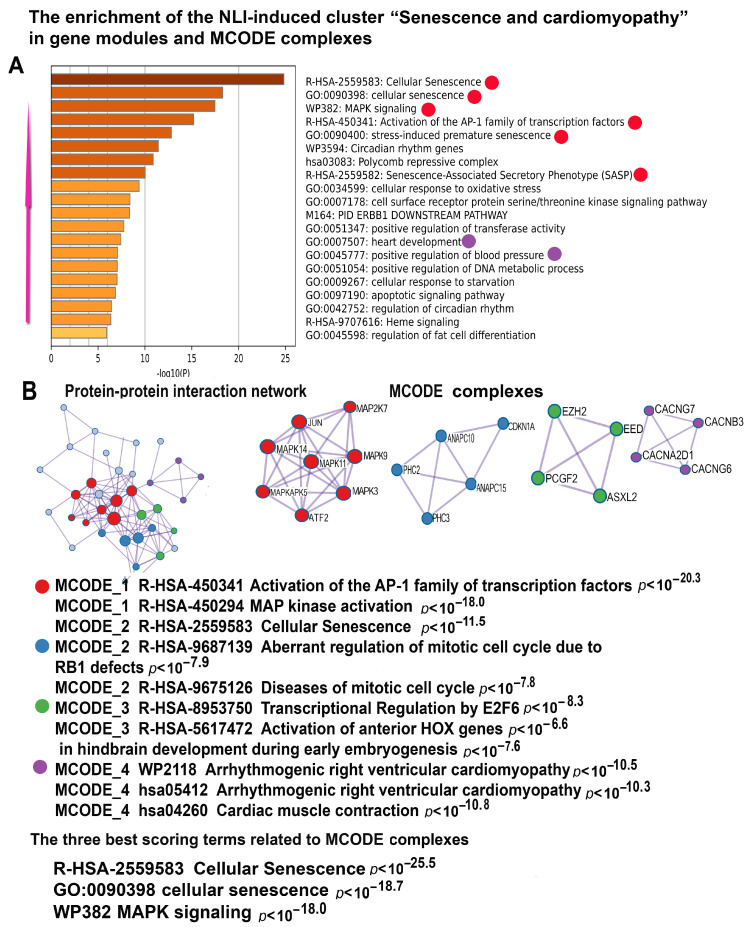
Enrichment of NLI-induced genes related to “Senescence and cardiomyopathy” cluster in gene pathways, processes, and molecular complexes. (**A**)—Histogram of gene modules and biological processes. The statistical significance of enrichment is shown on the X-axis (−log10(*p*)). (**B**)—Protein–protein interaction network and MCODE components (i.e., densely connected network components). The network and MCODE components were constructed based on physical interactions obtained from the STRING server (physical score > 0.4). The three most significant terms related to the MCODE components are highlighted. The coding by color squares reflects the MCODE components. The coding by color circles indicates the results of the MCODE component pathway and process enrichment analysis. The figure illustrates the association between NLI, premature heart senescence and cardiomyopathy.

Our data strongly support the well-established link between DNA damage in telomeric regions and the acceleration of cellular senescence [52,60,61]. Through our analysis, we identified a gene cluster significantly enriched in pathways regulating aging and senescence processes with high statistical significance. Key gene modules include “Cellular Senescence” (R-HSA-2559583, *p* < 10^−27^), “Stress Induced Premature Senescence” (GO: 0090400, *p* < 10^−13^), “MAPK Signaling” (WP382, *p* < 10^−16^), and “Senescence-Associated Secretory Phenotype (SASP)” (R-HSA-2559582, *p* < 10^−18^). These enriched pathways highlight the complex molecular interplay between NLI and senescence, thereby providing new insights into the triggers of these processes (Figure 9). The other signaling pathway, including “Activation of AP-family Transcription Factors” (Figure 9), is crucial in regulating cellular senescence. This pathway facilitates gene expression by promoting chromatin opening, a key driver of age-related chromatin remodeling [62]. The central regulators of this module include JUN and several members of the MAPK family (MAPK3, MAPK9, MAPK11, MAPK14) (Supplementary Table S2). This mechanism operates in synergy with the telomere-associated molecular complex enriched in ATP-dependent chromatin remodeling, which is essential for chromatin relaxation and structural reorganization found in the telomere-related cluster (Figure 9).

The investigation of senescence-related gene clusters using the MCODE algorithm identified a molecular complex related to cellular senescence, MAP kinase, and the activation of the AP-1 transcription factor family (Figure 9). In accordance, the three gene modules enriched for the upregulated genes with the highest significance include two modules related to cellular senescence and MAPK signaling. These results confirm the conclusions drawn from the enrichment analysis of the gene modules, emphasizing a link between NLI, cellular senescence, and chromatin opening (Figure 9*).*

The other signaling pathway, including “Activation of AP-family Transcription Factors” (Figure 9), is crucial in regulating cellular senescence. This pathway facilitates gene expression by promoting chromatin opening, a key driver of age-related chromatin remodeling [62]. The central regulators of this module include JUN and several members of the MAPK family (MAPK3, MAPK9, MAPK11, MAPK14) (Figure 9, Supplementary Table S2). This mechanism operates in synergy with the telomere-associated molecular complex enriched in ATP-dependent chromatin remodeling, which is essential for chromatin relaxation and structural reorganization found in the telomere-related cluster (Figure 9).

### 2.7. NLI Compromises Cardiac Function

Our data revealed several indications that NLI is associated with impaired heart function. It is evident from the enrichment of different gene clusters in gene modules and molecular complexes implicated in muscle contraction, heart rhythm, the regulation of blood pressure, epithelial to mesenchymal transitions, and others. Below, we describe these gene modules. In addition to identifying the senescence-related gene modules, the MCODE analysis of genes from the senescence-associated cluster revealed two key molecular complexes linked to heart dysfunction (Figure 9). The first complex, “Abnormal regulation of the mitotic cell cycle due to RB1 defects”, accelerates the transition from the G1 phase to the S phase in cardiomyocytes, driven by RB1 defects. This dysregulation can result in polyploidy, aneuploidy, and genomic instability, ultimately compromising cardiac function and potentially leading to dilated cardiomyopathy [63,64,65]. The second complex, “Arrhythmogenic right ventricular cardiomyopathy”, underscores the connection between NLI arrhythmias, further establishing the link between cellular aging markers and cardiac dysfunction. Together, these findings provide evidence for the interplay between NLI, aging pathways, and heart disease, highlighting potential mechanisms through which senescence impacts cardiovascular health.

**Figure 10:**
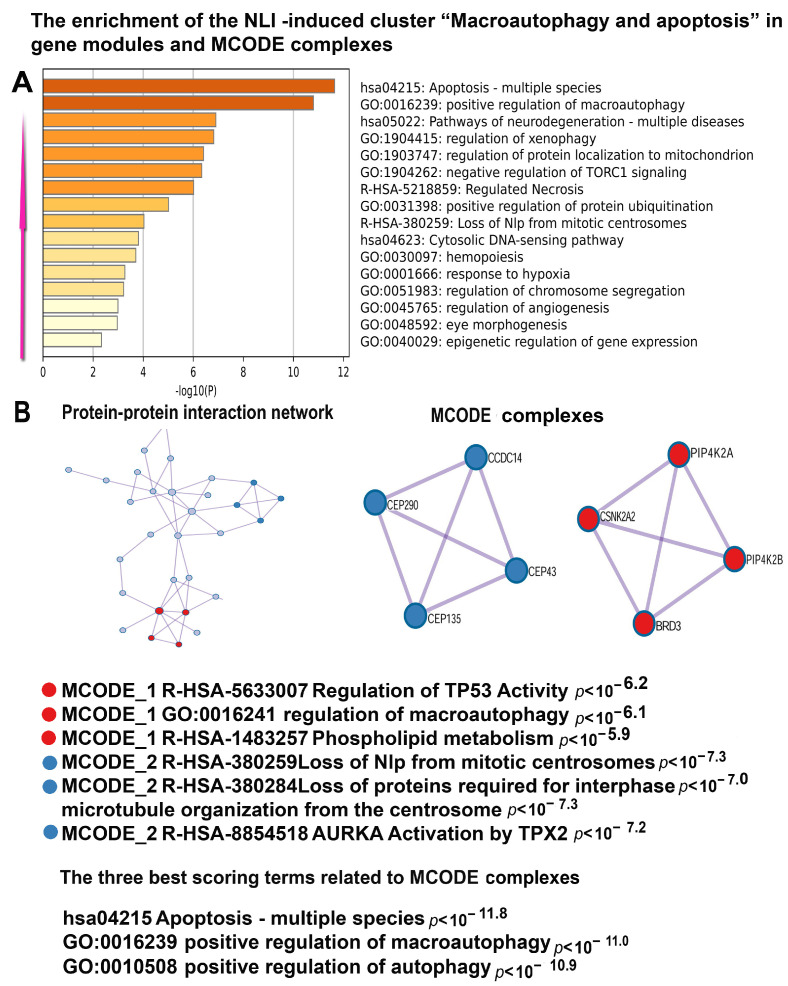
Enrichment of NLI-induced genes related to the “Macroautophagy” cluster in gene pathways, processes, and molecular complexes. (**A**)—Histogram of gene modules and biological processes. The statistical significance of enrichment is shown on the X-axis (−log10(*p*)). (**B**)—Protein–protein interaction network and MCODE components (i.e., densely connected network components). The network and MCODE components were constructed based on physical interactions obtained from the STRING server (physical score > 0.4). The three most significant terms related to the MCODE components are highlighted. The coding by color squares reflects the MCODE components. The coding by color circles indicates the results of MCODE component pathway and process enrichment analysis. The figure illustrates the association between NLI and long-term macroautophagy activation leading to cardiomyocyte atrophy.

In accordance with these results, our data revealed several more confirmations that NLI can compromise heart function. The most important of them is a cluster enriched in NLI-induced genes involved in signaling cascades related to apoptosis, necrosis, macroautophagy, and the negative regulation of mTORC1 signaling. The combination of the modules related to mTORC1 and autophagy points to strong macroautophagy activation because mTORC1 impairment is known to boost macroautophagy that can destroy essential cardiomyocyte components and lead to cell atrophy and apoptosis [66]. MCODE analysis identified a molecular complex related to macroautophagy and apoptosis (Figure 10).

**Figure 11:**
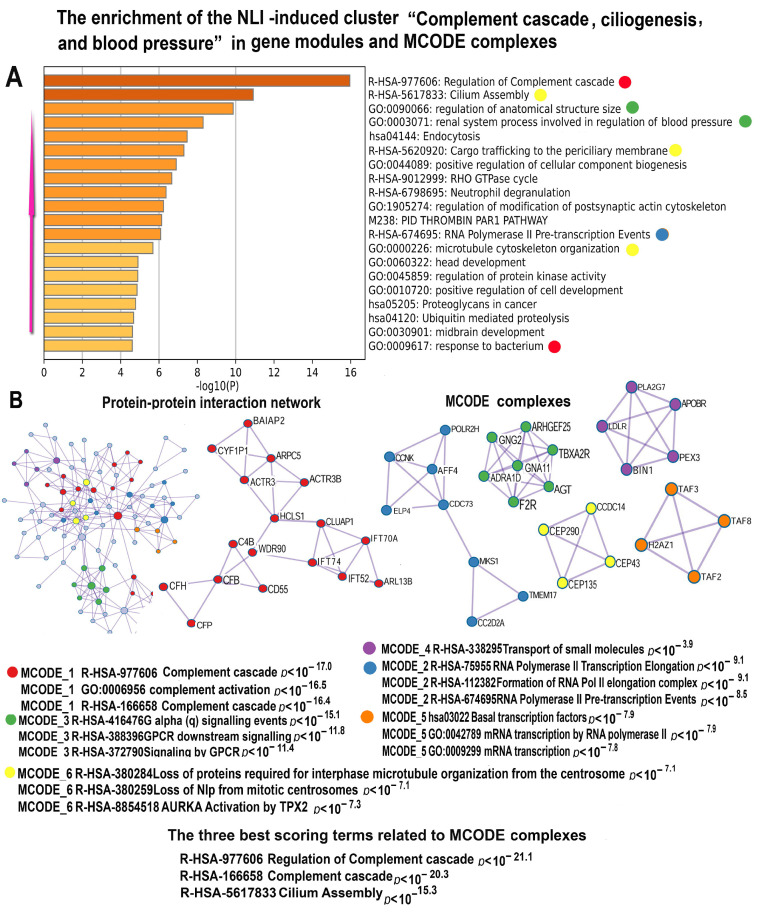
Enrichment of NLI-induced genes related to the cluster “Complement cascade, ciliogenesis and blood pressure” in gene pathways, processes, and molecular complexes. (**A**)—Histogram of gene modules and biological processes. The statistical significance of enrichment is shown on the X-axis (−log10(*p*)). (**B**)—Protein–protein interaction network and MCODE components (i.e., densely connected network components). The network and MCODE components were constructed based on physical interactions obtained from the STRING server (physical score > 0.4). The three most significant terms related to the MCODE components are highlighted. The coding by color squares reflects the MCODE components. The coding by color circles indicates the results of MCODE component pathway and process enrichment analysis. The figure illustrates the association between NLI and the long-term activation of pro-inflammatory cascade of complement, enhanced ciliogenesis, and defects of mitotic apparatus.

One more important NLI-induced gene cluster that can exert negative effect on cardiac function is the activation of pro-inflammatory response evident from the increased activity of gene modules related to a branch of innate immunity related to a cascade of complement participating in the release of pro-inflammatory cytokines (Figure 11). The activation of this cascade can enhance inflammation thereby increasing the risk of cardio-vascular diseases [67].

This cluster also is enriched in genes from modules “Renal system process involved in regulation of systemic arterial blood pressure”, Trombin pathway”, and “Regulation of anatomical structure size”. The other pathways that can play an important role in blood pressure elevation are presented by “Positive regulation of epithelial to mesenchymal transition”. The upregulation of this pathway induces cardiomyocyte narrowing and elongation thereby promoting delated cardiomyopathy [68]. It is also important to note that this cluster contains indications of the association between NLI and senescence seen from the upregulation of gene modules implicated in ciliogenesis. The enhanced ciliogenesis is known to promote apoptosis resistance and ciliogenesis.

MCODe analysis identified important molecular complexes confirming the impact of NLI to the disruption of mitotic apparatus and M-phase (Figure 11B). It is seen from the MCODE clusters enriched in the gene module “Loss of MLP from mitotic centrosome”. Thus, the results of the analysis of these two clusters (related to macroautophagy and complement) confirm that NLI accelerates heart aging and reduces its functional potential.

**Figure 12:**
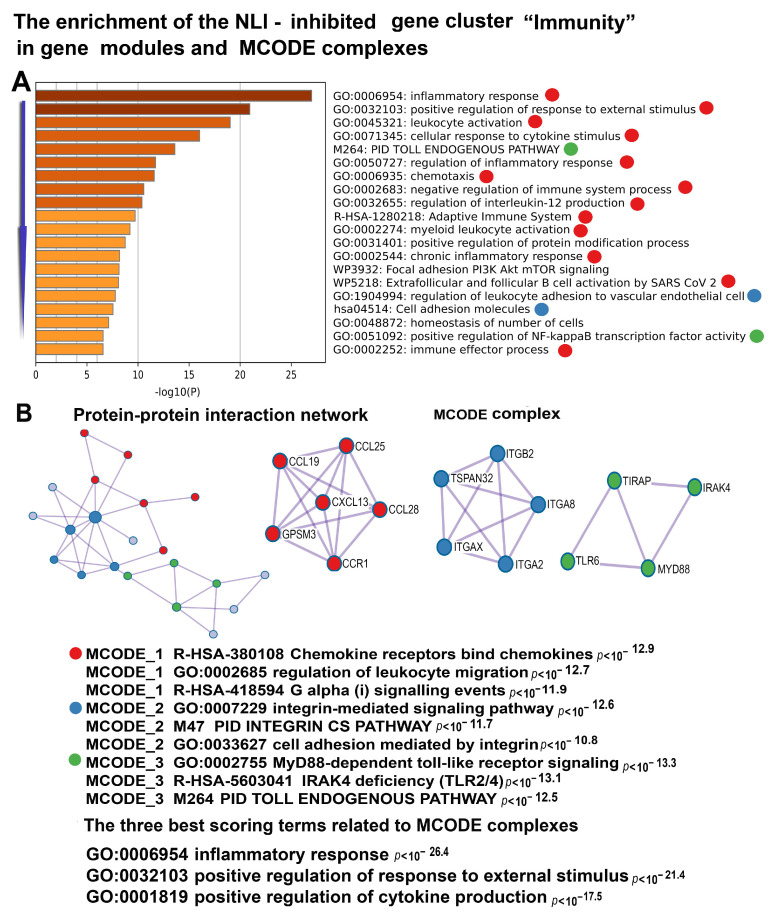
Enrichment of NLI-inhibited genes related to the cluster “Immunity” in gene pathways, processes, and molecular complexes. (**A**)—Histogram of gene modules and biological processes. The statistical significance of enrichment is shown on the X-axis (−log10(*p*)). (**B**)—Protein–protein interaction network and MCODE components (i.e., densely connected network components). The network and MCODE components were constructed based on physical interactions obtained from the STRING server (physical score > 0.4). The three most significant terms related to the MCODE components are highlighted. The coding by color squares reflects the MCODE components. The coding by color circles indicates the results of MCODE component pathway and process enrichment analysis. The figure illustrates the association between NLI and long-term impairment of acquired immunity.

It is important to note that the upregulation of gene modules related to the complement cascade is accompanied by the downregulation of genes involved in acquired immunity related to leucocyte (Figure 12). This result suggests that NLI causes long-term rearrangements in immune systems, as evident from the shift towards innate immunity.

**Figure 13:**
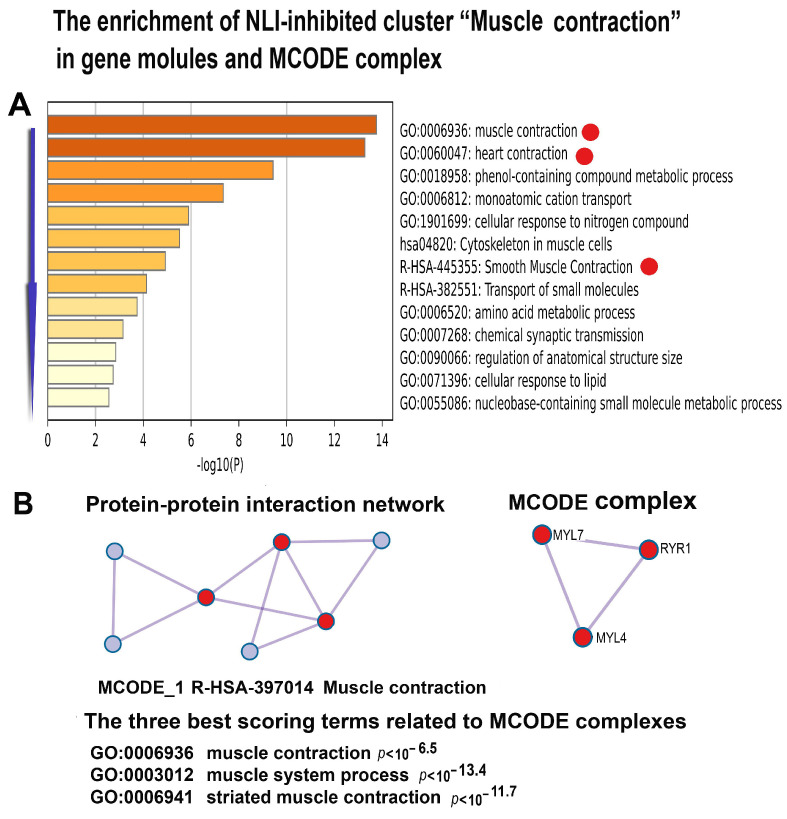
Enrichment of NLI-inhibited genes related to the “Muscle contraction” cluster in gene pathways, processes, and molecular complexes. (**A**)—Histogram of gene modules and biological processes. The statistical significance of enrichment is shown on the X-axis (−log10(*p*)). (**B**)—Protein–protein interaction network and MCODE components (i.e., densely connected network components). The network and MCODE components were constructed based on physical interactions obtained from the STRING server (physical score > 0.4). The three most significant terms related to the MCODE components are highlighted. The coding by color squares reflects the MCODE components. The coding by color circles indicates the results of MCODE component pathway and process enrichment analysis. The Figure illustrates the association between NLI and the long-term impairment of cardiac muscle contraction.

The NLI downregulated genes also cause the NLI-related decrease in heart function by a gene cluster involved in cardiac contraction enriched in gene modules “Muscle contraction”, “Smooth muscle contraction”, and “Heart contraction” and by the molecular complex enriched in genes related to “Muscle contraction” (Figure 13).

**Figure 14:**
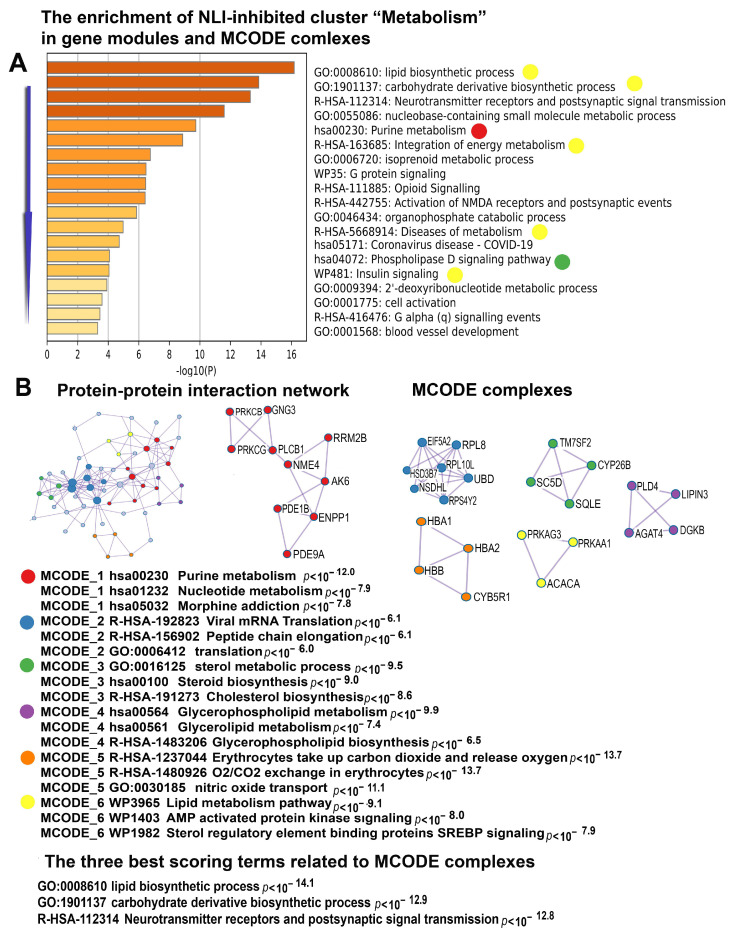
Enrichment of NLI-inhibited genes related to the “Metabolism” cluster in gene pathways, processes, and molecular complexes. (**A**)—Histogram of gene modules and biological processes. The statistical significance of enrichment is shown on the X-axis (−log10(*p*)). (**B**)—Protein–protein interaction network and MCODE components (i.e., densely connected network components). The network and MCODE components were constructed based on physical interactions obtained from the STRING server (physical score > 0.4). The three most significant terms related to the MCODE components are highlighted. The coding by color squares reflects the MCODE components. The coding by color circles indicates the results of the MCODE component pathway and the process-enrichment analysis. The Figure illustrates the association between NLI and the long-term impairment of energy metabolism.

These data are in good agreement with the manifestations of severe deprivation of energy metabolism seen from the cluster of downregulated genes enriched in genes belonging to pathways related to the metabolism of carbohydrates, lipids, and glycerophospholipids. Moreover, this cluster revealed the impairment of important energy-sensing pathways, including the signaling pathways operating via AMPK, SREBP, nitric oxide, insulin, and oxygen (Figure 14).

Overall, the results of this study highlight the profound impact of NLI on genomic and cellular integrity, revealing critical implications for cardiac health. By disrupting telomere maintenance, triggering chromatin remodeling, and impairing DNA repair pathways, NLI drives genomic instability, accelerates cellular senescence, and fosters the development of age-related cardiomyopathy. These effects are further exacerbated by disrupted energy metabolism, heightened inflammation, and diminished cardiomyocyte functionality, collectively undermining heart performance.

## 3. Discussion

Our study provides evidence confirming the link between neonatal lactose intolerance (NLI) and premature senescence and dysfunction of rat cardiomyocytes through mechanisms involving hyperpolyploidy, and transcriptomic changes indicative of DNA instability, telomere damage and fusion, chromatin remodeling, cell senescence, disturbed differentiation, inflammation, rearrangements of immunity, and deprivation of energy metabolism. NLI-associated heart remodeling at cellular and molecular levels was accompanied by the weakening of heart function: Echocardiography revealed structural and functional impairments in the LV of NLI-exposed rats, including increased end-diastolic and end-systolic diameters and corresponding volume enlargements. These changes were accompanied by a mild but statistically significant reduction of ejection fraction and fraction shortening pointing to maladaptive LV remodeling characteristic of early-stage cardiomyopathy. Our findings align with echocardiographic studies in adults with inflammatory bowel diseases, which report a link between gastroenteritis and LV dilation with reduced EF and FS [69,70]. They also support recent long-term follow-up data in pediatric patients, showing that Crohn’s disease and ulcerative colitis are associated with persistent subclinical LV systolic and diastolic dysfunction, even after disease remission [71].

The long-term consequences of NLI were further confirmed by survival analysis, which demonstrated a significant reduction in lifespan, highlighting the profound impact of early-life metabolic stress on overall longevity. This survival deficit suggests that NLI accelerates biological aging, likely through persistent oxidative stress, inflammation, and genomic instability, all of which contribute to premature cardiovascular decline. These findings are in line with human epidemiological data showing that early-life nutritional and inflammatory stressors increase the risk of age-related cardiovascular diseases, including heart failure, hypertension, and coronary artery disease [8,10,11].

One of the important findings of our study is that early postnatal development represents a critical period for heart formation because of high sensitivity to even subtle stress with potential lifelong cardiovascular consequences. Here, we demonstrated that systemic inflammatory stress during the neonatal period induces hyperpolyploidization of cardiomyocytes in various regions of the left ventricular myocardium in rats. This phenomenon points to a premature exit of cardiomyocytes from the mitotic cycle [26,27,28,72]. Instead of completing the final rounds of postnatal cell doubling typical to normal mitosis, cardiomyocytes cease division and begin to accumulate genomes [23]. The reduction in the number of complete cardiomyocyte divisions inevitably results in a lifelong decrease in their overall quantity [24].

Furthermore, genomic duplications significantly alter the properties of cardiomyocytes, as they mainly arise in this cell type in response to DNA damage in the telomeric regions and subsequent telomere fusion originating from their critical shortness [73,74,75]. These changes are accompanied by chromatin decompaction, an enhanced Warburg effect in metabolism, and the manifestation of fetal phenotypes, disturbed differentiation, YAP/TAZ signaling, and senescence [76,77,78,79,80,81,82,83,84]. Therefore, the excessive cardiomyocyte polyploidy can correlate with reduced regenerative capacity and impaired contractile efficiency [28,31,85,86,87,88]. This association underscores polyploidy’s dual role as an adaptive response to stress and a driver of cardiac aging, reflecting findings in human and animal models of cardiovascular disease.

Recent studies have demonstrated that cardiomyocyte DNA instability and telomere damage are associated with the postnatal metabolic transition from glycolysis to fatty acid metabolism and oxidative phosphorylation [89,90]. Given the synchrony of the postnatal metabolic shift, the accumulation of reactive oxygen species (ROS), and polyploidization, we hypothesized that additional ROS stress during this metabolic transition could amplify genome accumulation. This hypothesis is based on the observations that high levels of ROS production, designated as oxidative distress or damage, result in molecular damage, leading to genome alteration, cell cycle disturbance, and apoptotic death [91,92,93].

To verify this suggestion, we choose to develop and apply the experimental model of neonatal lactose intolerance because this condition is quite common in humans. It accompanies gastroenteritis of various etiologies, as these conditions often reduce the activity of the lactase enzyme thereby impairing the digestion of lactose [94]. This enzymatic deficiency leads not only to the well-recognized gastrointestinal symptoms of lactose intolerance but also to broader systemic effects, including inflammation [95] that increases ROS stress that in turn promotes polyploidy [73].

The systemic consequences of NLI are linked to the accumulation of metabolic byproducts from undigested lactose and the associated alterations in the gut microbiome [95]. These changes may disrupt gut homeostasis, fueling inflammatory responses that extend beyond the gastrointestinal tract [96]. Moreover, excessive lactose consumption can aggravate these effects by increasing galactose levels—a sugar with well-documented pro-aging properties at the cellular level [95,96].

Newborns are particularly vulnerable to NLI, as secondary lactase insufficiency often develops due to damage to the intestinal mucosa caused by infections or inflammatory conditions [97]. This heightened susceptibility underscores the critical need for effective management strategies during early development.

In preschool and school-aged children, lactose intolerance remains a prevalent issue. This is often the result of an overload of the lactase enzyme caused by excessive dairy consumption, highlighting the importance of dietary balance [98,99]. In more than half of the cases, this condition is asymptomatic, manifesting only as subclinical inflammation, which often goes unnoticed [100]. Thus, based on the data that polyploidization of human cardiomyocyte primarily occurs during childhood and early puberty [23,24], and that children are often susceptible to lactose intolerance with clinical and subclinical symptoms [98], the data obtained in this study can be important for uncovering hidden triggers of cardiovascular complications.

Beyond the association between NLI and a susceptible increase in cardiomyocyte polyploidy in various parts of the heart left ventricle, our data indicated that NLI induces global transcriptome changes by triggering complex processes at the cellular, molecular, and epigenetic levels, which have long-term detrimental effects on heart health.

One of the most important manifestations of NLI in transcriptome is the features of accelerated senescence. Our findings reveal that NLI induces genomic instability, characterized by telomere attrition and chromatin decompaction. This dual mechanism appears to drive polyploidization and cellular senescence, ultimately impairing cardiomyocyte differentiation and functionality [101,102,103]. These results align with previous research indicating that telomere damage and reduced chromatin integrity contribute to disturbed differentiation in cardiomyocyte, as well as age-related cellular decline and heightened susceptibility to cardiovascular diseases.

The activation of stress and DNA damage response pathways observed in our transcriptome-wide analysis further underscores the systemic nature of NLI impact. Notably, genes related to telomere maintenance and alternative lengthening mechanisms were significantly upregulated, suggesting compensatory but insufficient attempts to preserve genomic integrity under NLI-induced stress. This dysfunction likely stems from compromised energy metabolism, resulting in energy deficiency that disrupts normal cardiac function.

It is important to note that the findings of this study, based on genes with altered expression, complement and reinforce the results of our recent research, where we examined the long-term effects of NLI on switched-on or switched-off genes [31]. For example, the current data indicating telomere damage strengthen earlier findings that NLI activates certain branches of DNA damage response pathways. Again, the activation of aging-related signaling cascades aligns well with evidence of oxidative stress, inflammation, circadian clock activation, fibrosis, and weakened oxidative stress defenses. The link between these changes and aging is well documented. The observed disruption of energy metabolism and muscle contraction pathways found here complements and enhances the evidence of suppressed calcium and thyroid hormone signaling. Indeed, it is well established that thyroid hormone deficiency and the deprivation of calcium signaling dramatically deprives muscle contraction.

Notably, the comparison and integration of the data from this and a previous study has also provided a deeper understanding of NLI-associated immune system remodeling. Specifically, our data identified the activation of innate immunity and the suppression of adaptive immunity. The induction of innate immunity is evident from the activation of the complement system, innate immune pathways, and interferon biosynthesis in this study and [31]. The suppression of adaptive immunity is reflected in the reduced activity of gene modules related to leukocytes and pro-inflammatory cytokines. This strong concordance and complementarity between findings obtained through different approaches highlight the value of separately studying toggle (switch-on/off) genes alongside those with altered expression.

### 3.1. Practical Implications

Our study uncovers a newly recognized, highly sensitive, and critical window in postnatal heart development, during which even mild stressors—such as inflammation induced by neonatal lactose intolerance (NLI)—can trigger lifelong consequences. These include transcriptome-wide remodeling, the early onset of cardiomyocyte aging, progressive cardiac dysfunction, and even reduced lifespan. This discovery shifts the paradigm in pediatric and cardiovascular medicine, emphasizing that early-life metabolic and inflammatory stressors can permanently shape heart health. These findings underscore the urgent need for proactive screening, early diagnosis, and timely intervention in neonates and young children with suspected gastroenteritis and lactose intolerance to prevent irreversible cardiovascular damage.

A key practical implication of our research is the identification of novel, highly predictive biomarkers for long-term cardiovascular risk. We demonstrate that excessive cardiomyocyte polyploidization, telomere fusion and damage, the upregulation of aging, apoptosis, and autophagy markers, along with the downregulation of muscle contraction and energy metabolism markers, are not only hallmarks of NLI-induced stress but also early warning signs of cardiovascular aging. These molecular signatures provide a transformative tool for clinicians, enabling early risk stratification, personalized preventive care, and targeted therapeutic interventions years before clinical symptoms arise. Implementing these biomarkers into routine pediatric assessments could revolutionize early detection and significantly reduce the global burden of cardiovascular disease. Beyond diagnostics, our findings open new avenues for therapeutic innovation. We propose targeted interventions aimed at reducing oxidative stress, preserving telomere integrity, and modulating inflammatory responses as promising strategies to mitigate the long-term cardiovascular consequences of neonatal stress. This approach offers a precision-medicine framework for cardiovascular disease prevention from infancy. By elucidating these mechanisms, our study lays the groundwork for a fundamental shift in early-life clinical management, fostering the development of next-generation therapies and public health strategies to improve cardiovascular outcomes across the lifespan.

### 3.2. Perspectives

To validate whether the findings observed in rats apply to humans, longitudinal cohort studies should be conducted to track cardiovascular health over time in individuals with a history of neonatal lactose intolerance (NLI). Such research would provide critical insights into the long-term impact of early-life metabolic stress on heart function and overall health.

Further investigation is also needed to clarify the precise mechanisms through which NLI induces telomere instability and drives chromatin remodeling. Additionally, it is crucial to determine whether targeted interventions—such as antioxidant therapies, telomerase activators, or metabolic regulators—can counteract these effects and prevent long-term cardiovascular complications.

Given the systemic nature of the observed molecular and cellular changes, future studies should explore whether NLI contributes to aging-related dysfunction in other organs, including the liver, kidneys, and brain. Expanding research in this direction could help uncover the broader consequences of early metabolic stress on overall aging and disease susceptibility.

By advancing these research avenues, this study has the potential to lay the foundation for innovative preventive strategies and therapeutic interventions aimed at reducing the long-term burden of cardiovascular diseases linked to early-life metabolic stress.

## 4. Materials and Methods

### 4.1. The Animals Used in the Experiment

Adult rats (180–250 g) were obtained from the Rappolovo nursery. Females were paired overnight with age-matched males, and litters were standardized to eight pups (four males, four females) at birth. The control group was maintained under a 12 h light/dark cycle with free access to food and water [98].

### 4.2. Ethical Compliance Statement

This study adhered to the ethical principles outlined in the Declaration of Helsinki. All animal procedures were conducted in full compliance with the Animal Welfare Assurance (Assurance ID: F18-00380) established by the Institute of Cytology, Russian Academy of Sciences, to safeguard the well-being of animals bred on experimental farms and used in scientific research. Additionally, all experimental protocols followed the guidelines set forth in the Guide for the Care and Use of Laboratory Animals (1996) issued by the U.S. Department of Health and Human Services [104].

### 4.3. Study Design and Lactose-Enriched Diet

The experiment with lactose-enriched diet was the same as in our previous study [31]. Here, we provide a brief description. Lactose intolerance was induced in suckling rats by exceeding their intestinal lactase capacity. From postnatal day 8 to 21, pups were given lactose-dissolved water daily, a crucial period for cardiomyocyte maturation [105,106]. Lactose (Vecton, St. Petersburg, Russia) was administered according to age-related lactase activity: 8–16-day-old pups received twice their usual intake (342 mg), while 16–20-day-olds (43–54 g) received 1.3 times the normal amount (307 mg). The solution (1.5 mL distilled water, 37 °C) was delivered orally through flexible gavage tubes in 0.25 mL increments every hour for six hours. Pups readily consumed it. Controls received an equal volume of heated distilled water. Long-term effects were assessed at 140 days, with body weight recorded for 24 experimental and 24 control animals.

### 4.4. Estimation of Rat Survival Rate

To characterize the effect of NLI on rat survival, the Kaplan–Meier survival curves were plotted and log-rank test was applied to compare survival among groups with the aid of GraphPad Prism 8 scientific software (GraphPad Software Inc., La Jolla, CA, USA). For this purpose, 12 NLI rats and 12 control rats were followed up until they reached advanced age.

The natural lifespan of each animal was determined by recording the age at which it died. Comparison of Kaplan–Meier survival curves was performed through log-rank (Mantel–Cox) test and Median survival rate, as recommended in [107]. For the Kaplan–Meier survival plots, statistical significance was measured by log-rank (Mantel–Cox) test. A *p*-value < 0.05 was considered to be a significant difference.

### 4.5. Echocardiography

Prior to echocardiography, rats were weighted and anesthetized with peritoneum injection of the lowest effective dose of ketamine 37 mg/kg and xylazine 7 mg/kg, as was recommended by Stein and co-author, 2007. This amount is the minimum that is necessary for the induction and maintenance of adequate anesthesia during ultrasound examination (12–15 min). Then, the animal’s anterior chest was shaved and covered with pre-warmed echo transmission gel. Body temperature was maintained between 37 °C and 37.5 °C with a heating pad throughout the study. Rats were examined in the supine position. A Rascan ultrasound system (Ratex, Saint Petersburg, Russia) equipped with a 5–12 MHz linear array transducer or Acuson Sequoia system fitted with a 15-MHz linear-array transducer was applied. Two-dimensionally guided B-mode recordings were obtained from the parasternal long-axis view (PLAX). All measurements were averaged over five cardiac cycles. Left ventricular end-systolic (LVESd, mm) and end-diastolic (LVEDd, mm) diameters, as well as systolic and diastolic posterior wall thickness (LVPWs, mm) and (LVPWd, mm), and inter ventricular septum end-systolic and end-diastolic thickness (IVSs, mm) and (IVSd, mm) were measured from the B-mode tracings by using the leading-edge convention of the American Society of Echocardiography (Sahn, et al., 1978) [108]. End diastole was defined as the maximal LV diastolic dimension, and end systole was defined as the peak of posterior wall motion. LV fractional shortening (FS, %), ejection fraction (EF, %), end systolic volume (ESV, µL^3^), end diastolic volume (EDV, µL^3^), stroke volume (SV, µL), LV mass (g) were calculated according to the equations established by the American Society of Echocardiography.

ESV (µL^3^) = (7 × (LVESd)^3^)/(2.4 + LVESdEDV) (µL^3^) = (7 × (LVEDd)^3^)/(2.4 + LVEDd) (1)

EDV (µL^3^) = (7 × (LVEDd)^3^)/(2.4 + LVEDd) (2)

EF, % = ((EDV − ESV)/EDV) × 100%(3)

FS (%) = [(LVEDd − LVESd)/LVEDd] × 100%(4)

All measurements were made by a single experienced echocardiographer who was masked regarding animal group assignment. Overall, 12 lactose-treated and 12 control animals were investigated for each age group.

### 4.6. Cardiomyocyte Isolation

Cardiomyocytes were isolated as described in [109,110]. Rats were euthanized via intraperitoneal injection of ketamine (100 mg/kg) and xylazine (10 mg/kg). Hearts were excised and subjected to a modified enzymatic retrograde coronary perfusion. Initially, they were perfused with Tyrode’s solution for 3 min, followed by a low-Ca^2^⁺ solution for 5 min. Enzymatic digestion was then performed for 9 min using Tyrode’s buffer containing collagenase II (1.1 mg/mL) and hyaluronidase (0.5 mg/mL). The left ventricle (LV) was dissected, and small tissue fragments (~15 mg each) were collected from three distinct regions: (1) the apex, where contractions initiate to propel blood into systemic circulation; (2) the LV base, positioned beneath the left atrium; and (3) the mid-interventricular septum, which ensures proper blood flow direction. Tissue fragments were finely minced and gently resuspended to release cells. Digestion was halted with a buffer containing 10% calf serum and 12.5 μM CaCl₂ [110]. The cell suspension was filtered through a 200 μm nylon mesh, sedimented by gravity, and examined under a phase-contrast microscope for concentration and integrity. For further analysis, cell smears were prepared by placing five drops of suspension on glass slides, air-dried, and fixed with absolute methanol.

### 4.7. Staining and Assessment of Cardiomyocyte Ploidy and DNA Instability

Cardiomyocyte smears were stained with Hoechst 33258 (20 µg/mL, AxisPharm, San Diego, CA, USA) for 15 min to quantify DNA content. Imaging analyses, including cytometry and morphometry, were conducted using a Zeiss Axioskop microscope (Carl Zeiss AG, Oberkochen, Germany) with a CCD VarioCam digital camera (PCO Computer Optics GmbH Infrarotsensorik und Messtechnik, Dresden, Germany),and ImageJ software (version 1.54k). Splenocytes from age-, sex-, and weight-matched control animals served as the diploid standard. To assess cell ploidy, photometric analysis was performed on at least 500 cardiomyocytes from each of the three left ventricular fragments, totaling 1500 cells per sample. This study included 24 experimental and 24 control animals. In 3-week-old rats, fewer than 1% of cardiomyocytes were in the S-phase of the cell cycle [100]. To distinguish S-phase cells from polyploid cells, those with ploidy deviating more than 10% from multiples of 2n were excluded. The average genome content per cardiomyocyte was calculated using the formula:

PLD = ∑i × ni

where PLD is the mean number of genomes per cell and ni is the fraction of cardiomyocytes of the i-th ploidy class.

DNA instability in cardiomyocytes was assessed through the detection of nuclear bridges [ 66] and the frequency of aneuploid cells, characterized by nuclei containing an abnormal number of chromosomes. A total of 500 cells were examined for each of the 24 experimental and 24 control animals from three regions of the left ventricle (tip of the apex, base, and septa).

### 4.8. Assessment of Telomere Fusion

To evaluate telomere fusion, we quantified the number of dumbbell-shaped nuclei, characterized by nuclei connected to each other as recommended in Aix et al., 2023 [73]. For this purpose, smears of isolated cardiomyocytes were stained with aqueous solutions of Hoechst 33258, as described in Section 4.5. The number of dumbbell-shaped nuclei was assessed in 1000 cells from each of three control and three experimental animals (in the three regions of the left ventricle).

### 4.9. mRNA Sequencing and Analysis

mRNA sequencing was conducted by Genotek Co. (Moscow, Russia), according to the method, described in detail in [31]. Briefly: Total RNA was extracted from 10–15 mg of apical left ventricular myocardium using the PureLink RNA Mini Kit (Ambion, Life Technologies, Austin, TX, USA) and stored at −80 °C. mRNA was isolated using magnetic beads (Sileks, Badenweiler, Germany), and cDNA libraries were prepared with the NEBNext^®^ mRNA Library Prep Kit for Illumina (New England Biolabs, Ipswich, MA, USA). Library quality was assessed on a Bioanalyzer 2100 (Agilent Technologies, Santa Clara, CA, USA). Sequencing was performed on an Illumina HiSeq2500 (100 nucleotides, rapid run mode). Next-generation sequencing (NGS) was conducted for three biological samples each from control and lactose-treated groups. Reads were trimmed with Trimmomatic and mapped to the canonical rat transcriptome from RefSeq using Bowtie 2 (v2.5.0, “very sensitive” preset). Differential expression was analyzed with the “limma” package in R (version 3.54.0) optimized for whole-transcriptome analysis.

### 4.10. Identification of Quantitatively Deregulated Genes

To identify quantitatively deregulated genes, we focused on those expressed in both experimental and control groups, excluding switched-on and switched-off genes analyzed previously [31]. These quantitatively deregulated genes were selected from the largest cluster in the upper-right area of the two-dimensional histogram (Figure 4). For stringent pathway analysis, we applied a multistep signal-to-noise filtration approach based on protein–protein interaction (PPI) networks to minimize false positives and highlight robust effects. Using the STRING database [111], we identified highly reliable PPIs for genes with expression differences at *p* < 0.05, analyzing up- and downregulated genes separately. This yielded 2102 differentially expressed genes (DEGs): 1120 upregulated and 982 downregulated. Next, we identified key regulators (hubs) by selecting genes encoding proteins with more than five interactions, refining the dataset to 352 upregulated and 349 downregulated hub genes. Functional clustering, performed with the Glay method in Cytoscape, revealed four major clusters (>20 genes each) for upregulated hubs and two for downregulated ones [112,113,114]. These clusters were analyzed using the Metascape server [38], which integrates physical PPI enrichment and Molecular Complex Detection (MCODE) to identify significant molecular modules. Analyzing entire clusters, rather than individual genes, reduced spurious associations by considering combined statistical significance.

### 4.11. Statistical Analysis

Statistical analyses were conducted using GraphPad Prism 8, Statgraphics Centurion 18, the R statistical package, and the Metascape server [38]. The significance of differences between experimental and control groups is presented in each figure or described in the corresponding caption. Rigorous statistical validation ensures the robustness and reliability of the reported findings.

## 5. Conclusions

Our study established a clear link between neonatal lactose intolerance (NLI) and premature senescence of cardiomyocytes in rats, driven by mechanisms involving hyperpolyploidy, genomic instability, and transcriptomic alterations. These findings emphasize the increased vulnerability of early postnatal development to even subtle stressors, with lifelong cardiovascular implications. One of the important discoveries of our study is that NLI-induced systemic inflammatory stress during the neonatal period disrupts the normal mitotic cycle of cardiomyocytes. This leads to hyperpolyploidization, premature cell cycle exit, and incomplete cell doubling. This inevitably reduces cardiomyocyte numbers and impairs their contractile efficiency and regenerative capacity. DNA damage in telomeric regions, telomere fusion, and chromatin remodeling exacerbate these changes, contributing to impaired differentiation and cardiac aging. The metabolic shift from glycolysis to fatty acid metabolism and oxidative phosphorylation during early postnatal life exacerbates NLI effects, amplifying reactive oxygen species (ROS) stress and contributing to polyploidization and telomere attrition.

Transcriptomic analysis reveals insufficient compensatory mechanisms, such as telomere maintenance and DNA repair, failing to prevent genomic instability under NLI-induced stress. NLI also impairs energy metabolism and gene modules related to muscle contraction, resulting in energy deficiency and impaired heart function. Immune system remodeling further compounds these effects, with innate immunity activation and the suppression of adaptive responses, potentially impacting long-term cardiovascular health. The alignment of our findings with previous research reinforces that NLI triggers aging-like processes, including oxidative stress, inflammation, and fibrosis. This study underscores NLI as a systemic disorder with far-reaching effects beyond gastrointestinal symptoms, highlighting the need for early management to prevent lifelong cardiovascular risks. It lays a solid foundation for exploring the hidden mechanisms driving cardiac dysfunction linked to neonatal stress and metabolic disorders.

## Data Availability

Raw data may be provided by reasonable request. The authors confirm that the data supporting the findings of this study are available within the paper and its Supplementary Materials.

## Supplementary Materials

The following supporting information can be downloaded at: https://www.mdpi.com/article/10.3390/ijms26041584/s1.
