# Supplementary material for: Transcriptome-Wide Insights: Neonatal Lactose Intolerance Promotes Telomere Damage, Senescence, and Cardiomyopathy in Adult Rat Heart
Source: Int J Mol Sci. 2025 Feb 13;26(4):1584. doi: 10.3390/ijms26041584 (PMC11855832; doi:10.3390/ijms26041584)
Supplement: Supplementary file 1 [file ijms-26-01584-s001.zip › Supplementary Table S1.pdf]

**Supplementary Table S1.**

## Long-term effects of early postnatal lactose enriched diet on physical parameters and left ventricular morphology and function

| 12 weeks old animals<br>20 weeks (140 days) | Control (n=12) | The experiment (n=12) | Body weight adjusted values for control (n=12), (%) | Body weight adjusted values for the experiment (n=12), (%) |
|---|---|---|---|---|
| Body weight, g | 249±9.45 | 198±7.44** | ----- | ----- |
| Heart wight, mg | 1091±11.2 | 884±6.1** | 0.43±0.052 | 0.44±0.046 NS |
| LV mass (free wall+septa), mg | 881±25 | 806±24* | 0.354±0.032 | 0.41±0.027* |
| IVSs, mm | 2.64±0.09 | 2.6±0.12 NS | 0.010±0.002 | 0.012±0.002 NS |
| IVSd, mm | 1.7±0.09 | 1.6±0.11 NS | 0.006±0.001 | 0.010±0.001 NS |
| LVPWs, mm | 2.7±0.12 | 2.15±0.10 * | 0.010±0.002 | 0.010±0.001 NS |
| LVPWd, mm | 1.7±0.08 | 1.41±0.10 NS | 0.006±0.001 | 0.007±0.001 NS |
| LVEDs, mm | 3.4±0.21 | 4.7± 0.4** | 0,013±0.001 | 0.018±0.001* |
| LVEDd, mm | 6.41±0.51 | 7,6 ±0.38** | 0.025±0.003 | 0.038±0.003** |
| EF,% | 77,3±6.1% | 66,7±6.1%** | ----- | ----- |
| EDV, $\mu l^3$ | 209.26±18 | 309.28±12.3** | 0,83±0.056 | 1,55±0.045** |
| FS,% | 46±3.1 | 37±2.8** | | |
| ESV, $\mu l^3$ | 47.3±4.1 | 102.36±6.2** | 0,19±0.02 | 0,51.02** |
| LV mass, mg (calculated) | 681.6±29 | 686,24±28 NS | 0.271±0.042 | 0.346±0.034 NS |

*** - p**<0.05 for the difference with control; **** - p**<0.01 for the difference with control; **NS-** no difference with control. **LVESd** -LV end-systolic diameter; **LVEDd**-LV end-diastolic diameter;

**LVPWs** -LV posterior wall thickness in systola; **LVPWd** -LV posterior wall thickness in dyastola; **LVPWd** -LV posterior wall thickness in diastola; I**VSs**-end-systolic thickness; and **IVSd** end-diastolic thickness; **EF -** ejection fraction; **ESV** - LV end systolic volume; **EDV** -LV end diastolic volume;
